# Transvaginal repair for ureteral injuries caused by sutures: A case report

**DOI:** 10.1097/MD.0000000000045812

**Published:** 2025-11-14

**Authors:** Jimiao Huang, Yan Lin, Junfeng Li, Hang Lin, Lan Zhen, Lingsi Chen, Jianguang Ji, Xiangqin Zheng, Huan Yi

**Affiliations:** a Department of Gynecologic Oncology, Fujian Maternity and Child Health Hospital College of Clinical Medicine for Obstetrics and Gynecology and Pediatrics, Fujian Medical University, Fuzhou, Fujian, China; b Medical Research Center, Fujian Key Laboratory of Women and Children’s Critical Diseases Research, Fuzhou, China; c Faculty of Health Sciences, University of Macau, Taipa, Macau SAR, China; d Cancer Center, Faculty of Health Sciences, University of Macau, Taipa, Macau SAR, China.

**Keywords:** hysterectomy, iatrogenic complications, minimally invasive ureteral surgery, ureteral injury

## Abstract

**Rationale:**

Ureteral injury from sutures during total hysterectomy is a relatively uncommon but potentially severe complication that can lead to serious sequelae such as ureterovaginal fistula or renal insufficiency. The management of such injuries remains challenging, and there is a need for effective and minimally invasive approaches to mitigate complications and enhance patient recovery. This study aims to introduce a novel minimally invasive alternative – transvaginal suture removal and ureteral stenting under local anesthesia – and to evaluate its efficacy in reducing complications and improving patient outcomes.

**Patient concerns:**

A 49-year-old female experienced mild lumbar pain on the fourth day after total laparoscopic hysterectomy for uterine fibroids and adenomyosis. She had no prior kidney issues but developed postoperative right low back pain, microscopic hematuria, and ultrasound evidence of right hydronephrosis and ureteral dilation.

**Diagnoses:**

The patient was diagnosed with iatrogenic ureteral injury due to sutures, confirmed by ultrasound and surgical video review showing the right ureter partially sutured to the vaginal stump.

**Interventions:**

We performed transvaginal suture removal and ureteral stenting under local anesthesia. This minimally invasive approach avoided general anesthesia and secondary laparoscopic surgery.

**Outcomes:**

The procedure was successful, with smooth suture removal and stent implantation. The patient’s symptoms significantly improved on the second postoperative day. Follow-up at 1 month revealed no discomfort, and the ureteral stent was removed 3 months later.

**Lessons:**

This case highlights transvaginal suture removal as a novel, minimally invasive treatment for iatrogenic lower ureteral injuries. This approach may be performed by gynecologists under local anesthesia, potentially preventing severe complications and reducing patient discomfort and costs.

## 1. Introduction

Iatrogenic ureteral injuries are potential complications following total hysterectomy, with suture-induced ureteral injury being a common cause.^[1]^ Prompt intraoperative repair of ureteral injuries helps patients avoid secondary surgery and prevent related complications.^[2]^ However, intraoperative detection of ureteral injuries is challenging, with approximately 60% of patients not diagnosed in a timely manner.^[3]^ When serious complications arise, such as ureterovaginal fistula, sepsis, ureteral obstruction, or renal insufficiency, they can lead to physical and psychological distress for the patient.

Conventional treatments for postoperative detected ureteral injuries resulting from sutures typically involve laparoscopic suture removal from the vaginal stump and subsequent J ureteral stent implantation during cystoscopy. However, this approach necessitates tracheal intubation, general anesthesia, and a secondary laparoscopic procedure, leading to increased patient discomfort, higher hospitalization costs, and potential clinical complexities. These limitations highlight the need for alternative, less invasive methods that can reduce patient morbidity and healthcare costs.

In our study, we established that transvaginal removal of vaginal stump sutures offers a novel therapeutic approach for iatrogenic injuries to the proximal vesical ureter. This procedure, conducted under local anesthesia, can be independently performed by a gynecologist with minimal patient impact. This minimally invasive approach effectively mitigates the risk of severe complications, particularly distal ureteral injuries, while alleviating patient discomfort and reducing medical costs, thereby enhancing overall clinical outcomes.

## 2. Case presentation

A 49-year-old female underwent a total laparoscopic hysterectomy at our hospital due to multiple uterine fibroids and adenomyosis. The operation lasted for 2 hours, and the intraoperative assessment showed normal ureteral peristalsis. After the procedure, the patient did not experience fever or abdominal pain. However, on the morning of the fourth day postoperation, she reported mild lumbar pain. During the ward round, a thorough physical examination was conducted, and a urinary system ultrasound was performed. After carefully reviewing the examination reports and the surgical video, the diagnosis of iatrogenic ureteral injury due to sutures was confirmed for this patient. The diagnosis was supported by the following observations: absence of prior kidney issues or complaints, but postoperative right lateral low back pain; detection of microscopic hematuria; On ultrasound imaging, right hydronephrosis was identified, with an anteroposterior diameter of the renal collecting system of approximately 1.85 cm. Additionally, a dilated right ureter expanded to a maximum internal diameter of about 1.56 cm. A hyperechoic shadow was visible 1.5 cm from the right ureteral vesical orifice, which was considered to be due to suture, as depicted in Figure S1, Supplemental Digital Content, https://links.lww.com/MD/Q605, and review of the surgical video revealed partial suturing of the right ureter to the right lateral side of the vaginal stump, with the right ureter showing stenosis about 1.5 cm from the bladder orifice, as shown in Video 1.

**Figure 1.**
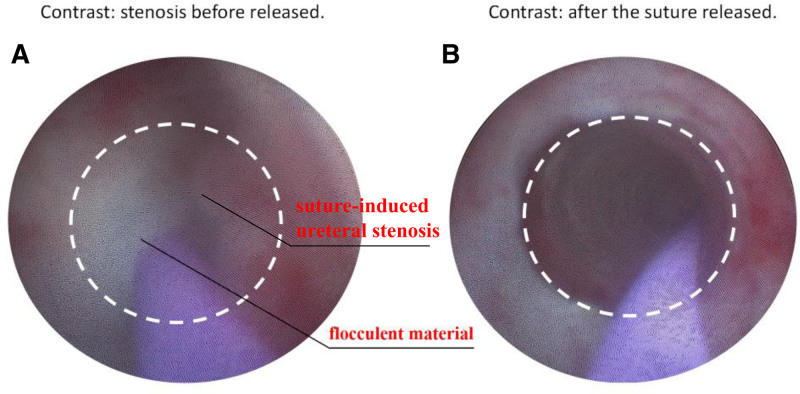
(A) Stenosis was detected 1.5 cm above the right ureteral opening, with a large amount of flocculent material obstructing the lumen, making it challenging to place the stent. (B) Ureteroscopy and stent implantation were successfully performed after releasing the right vaginal sutures. During the procedure, flocculent material was observed flowing out of the ureter, and the ureter was restored to patency.

The patient was scheduled for surgery that afternoon, with an initial plan to perform a cystoscopy and right ureteral stenting. However, during the cystoscopy, we observed a slit-like appearance of the right ureteral orifice with weakened urine flow and no significant bleeding. The stent implantation process was complicated by a significant obstruction within the ureter caused by a large accumulation of flocculent material, as depicted in Figure 1A and Video 1. Despite our attempts to change the guide wire and adjust its insertion direction, the procedure still failed. We finally opted to proceed with transvaginal suture removal and ureteral stenting for this patient due to several factors: 1) the site of injury located in the lower ureter; 2) a minimal risk of vaginal bleeding considering prior coagulation of uterine vessels and capillaries within 24 hours; 3) the simplicity and ease of performing under local anesthesia and minimizing patient discomfort. After consulting with the anesthesiologist, we administered local anesthesia using 1% lidocaine for a fan-shaped infiltration along the right side of the vaginal stump, reaching 3 to 5 mm beneath the mucosa. The total volume was kept under 20 ml, and we confirmed the analgesic effect after a 5-minute wait. Using a bivalve vaginal speculum (model: small), we fully exposed the vaginal stump and irrigated it with normal saline. We then created a traction fixation on the right side with a 3-0 absorbable suture. Based on preoperative ultrasound and intraoperative palpation, we located the suture in the upper 1/3 of the stump’s right side. We performed a sharp dissection 2 to 3 mm deep to expose the suture (1-0 absorbable), using bipolar electrocoagulation (25 W) for any minor bleeding. With microscissors, we carefully cut the suture near the knot and gently removed it with forceps, ensuring no ureteral mucosal tearing. After confirming no clear fluid leakage from the stump, indicating no fistula, we prepared for ureteral stent placement. We inserted an F9 rigid ureteroscope through the urethra and advanced a 0.035 inch super-slippery guide wire through the affected ureteral orifice, extending it 5 cm beyond the damaged area. Along the guide wire, we placed a 26 cm F4.7 double-J stent, ensuring the proximal end was in the renal pelvis and the distal end in the bladder. After confirming correct placement with ultrasound, we removed the guide wire (Fig. 1B). We then placed a gelatin sponge (2 cm × 3 cm) and a sterile gauze strip (10 cm in length) on the vaginal stump to apply pressure for hemostasis, which were removed after 24 hours. The patient was given cefmetazole for 3 days as prophylactic treatment against infection. The patient experienced a notable reduction of postoperative back pain with a favorable prognosis. The specific timeline details can be found in Figure S2, Supplemental Digital Content, https://links.lww.com/MD/Q605. The patient acknowledged our timely intervention and provided verbal informed consent for reporting this case during a 1-month postoperative telephone follow-up. Three months after the surgery, the patient returned to the hospital for follow-up. The right-sided back pain had completely resolved. Follow-up ultrasound examination revealed that the double-J stent was in the correct position, and the ureter and hydronephrosis had normalized (Fig. S3, Supplemental Digital Content, https://links.lww.com/MD/Q605). The double-J stent was successfully removed during this hospital stay.

## 3. Challenges and solutions

During the procedure, we encountered 2 significant challenges and implemented corresponding solutions.

**Challenge 1:** Significant resistance was encountered when passing the guide wire through the ureteral stricture during stent placement. The stricture was caused by suture compression, leading to mucosal edema in the ureteral segment. The resistance was graded as level II, meaning that slight force was needed but there was no clear breakthrough sensation – an issue that could lead to guide wire buckling or ureteral perforation.

**Solutions:** Changing the guide wire type: We replaced the super-slippery guide wire with a hydrophilic coated guide wire (e.g., Terumo guide wire) to reduce mucosal friction.

Ureteral decompression: After the initial attempt to pass the guide wire failed, we removed the suture from the right vaginal stump to decompress the ureter and then reattempted guide wire passage.

**Challenge 2:** There was a risk of active bleeding from the vaginal stump after suture removal due to its rich vascular supply.

**Solutions:** Immediate hemostasis: We used bipolar electrocoagulation (power 25W) to achieve hemostasis at the bleeding site.

Postoperative bleeding prevention: We placed a gelatin sponge (2 cm × 3 cm) and a sterile gauze strip (10 cm in length) on the vaginal stump to apply pressure for hemostasis, which were removed after 24 hours.

## 4. Discussion

This case highlights the successful management of an iatrogenic ureteral injury using a minimally invasive approach. The incidence of ureteral injury is relatively common in benign and malignant gynaecological surgery, and studies have reported that iatrogenic ureteral injuries occur during gynecological surgery, with an incidence of 52% to 80%.^[4,5]^ If not detected in time, these injuries can cause severe complications to the patient and cause unnecessary disputes. Therefore, early detection of patients’ symptoms and minimally invasive interventions are important for their prognosis. This article provides a new idea for the treatment of lower ureteral injuries by describing the method of transvaginal suture removal + ureteral stent implantation, which we hope will be applied by more gynecologists.

Currently, the management of ureteral injuries is determined by the severity and location of the injury. For short-segment injuries in the proximal or mid-ureter, laparoscopic uretero-ureterostomy is often performed.^[6]^ When renal pelvis access is limited by fibrosis or ischemia, lower pole uretero-calycostomy is preferred.^[7]^ Distal ureteral injuries, due to their proximity to the bladder, are prone to ischemic complications and are usually managed with ureteroneocystostomy.^[8]^ If significant ureteral length is lost, making tension-free reimplantation difficult, a psoas hitch or Boari flap can assist in ureteroneocystostomy.^[7]^ For ureteral injuries caused by sutures, traditional treatment involves laparoscopic suture removal from the vaginal stump followed by J ureteral stent implantation during cystoscopy. This approach has the advantage of addressing injuries at different locations but is more traumatic and requires secondary anesthesia, which can lead to patient dissatisfaction and complaints. Research indicates that laparoscopic procedures typically necessitate general anesthesia with endotracheal intubation, with an average anesthesia duration of approximately 119 minutes.^[9]^ Furthermore, around 80% of patients experience nausea and about 30% suffer from vomiting following surgery.^[10]^ In contrast, our technique uses vaginal local infiltration anesthesia with 1% lidocaine, with an anesthesia operation time of ≤ 5 minutes, and no anesthesia-related adverse reactions in this case, avoiding the potential impact of general anesthesia on cardiopulmonary function, especially suitable for elderly patients with underlying diseases. Regarding surgical trauma and recovery efficiency, traditional procedures require the establishment of 3 to 4 laparoscopic operating ports (diameter 5 to 10 mm), with a visual analogue scale score for postoperative abdominal wall pain of 2.62 ± 2.28 at 24 hours, often accompanied by shoulder pain.^[11]^ Patients are typically unable to get out of bed within 24 hours after surgery. Our technique, on the other hand, involves no abdominal wall incision, only local operation at the vaginal stump, allowing the patient to get out of bed and move around 6 hours after surgery, with a visual analogue scale score of 1.0 for vaginal stump pain the next day. From an economic perspective, traditional laparoscopic procedures are typically associated with longer surgery times, higher costs, and extended hospital stays. In contrast, our technique offers significant advantages by reducing surgery time, shortening hospital stays, and lowering overall costs. This approach not only enhances resource efficiency but also aligns well with the principles of enhanced recovery after surgery.^[12]^ To our knowledge, this is a novel treatment approach for iatrogenic injuries to the proximal bladder segment of the ureter, offering potential to prevent severe complications and mitigate medical disputes. The procedure is straightforward and can be performed in general hospitals, making it especially suitable for non-tertiary centers where specialized surgical expertise may be limited. Since it does not require special equipment or instruments, it can be easily adopted in various healthcare settings. For lower ureteral segment injuries suspected to be caused by sutures, this method can be considered for treatment after a safety assessment. However, the applicability of this approach is limited to lower ureteral segment injuries caused by sutures and ureteral orifice stenosis due to sutures. Additionally, the small sample size restricts the generalizability of our findings. Future studies with larger cohorts are needed to further validate the procedure and extend its benefits to a broader patient population.

In conclusion, promptly recognizing the patient’s initial complaints and thoroughly reviewing both the surgical video and ultrasound aided in early detection. Our approach of transvaginal suture removal may provide a novel alternative for managing iatrogenic ureteral injuries, potentially enhancing the efficacy of ureteral stent implantation. However, further research is needed to fully understand the long-term outcomes of this approach. A multi-center study could be proposed to validate the effectiveness and safety of this technique. We hope that our less invasive and patient-friendly approach could gain wider adoption among gynecologists, thereby significantly enhancing clinical practice and improving patient outcomes.

## Acknowledgments

We would like to acknowledge all the members of the Gynaecological Oncology Department in our hospital.

## Author contributions

**Conceptualization:** Huan Yi.

**Data curation:** Yan Lin, Lingsi Chen, Huan Yi.

**Formal analysis:** Jimiao Huang, Junfeng Li, Xiangqin Zheng, Huan Yi.

**Funding acquisition:** Huan Yi.

**Investigation:** Jimiao Huang, Hang Lin, Lan Zhen, Xiangqin Zheng, Huan Yi.

**Methodology:** Jimiao Huang, Yan Lin, Lan Zhen.

**Project administration:** Jianguang Ji.

**Resources:** Yan Lin, Hang Lin, Lingsi Chen, Huan Yi.

**Software:** Jimiao Huang, Jianguang Ji.

**Supervision:** Jianguang Ji, Huan Yi.

**Visualization:** Xiangqin Zheng.

**Writing – original draft:** Jimiao Huang.

**Writing – review & editing:** Junfeng Li, Jianguang Ji, Huan Yi.

## Supplementary Material


