# Supplementary figures and images for: Transvaginal repair for ureteral injuries caused by sutures: A case report
Source: Medicine (Baltimore). 2025 Nov 14;104(46):e45812. doi: 10.1097/MD.0000000000045812 (PMC12622740; doi:10.1097/MD.0000000000045812)

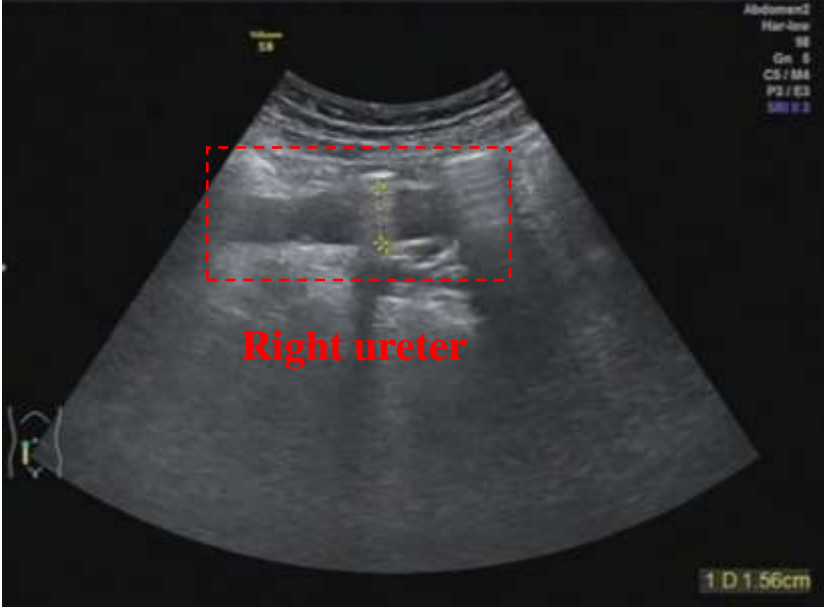

A

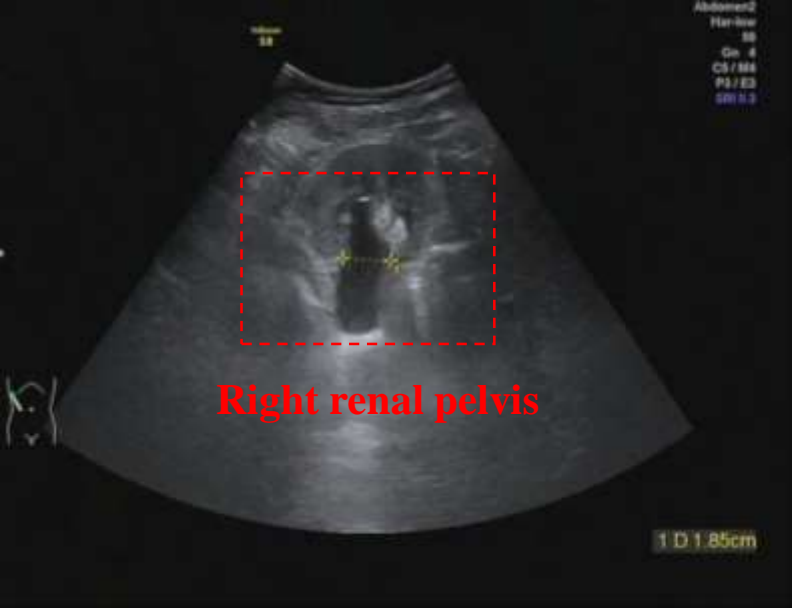

B

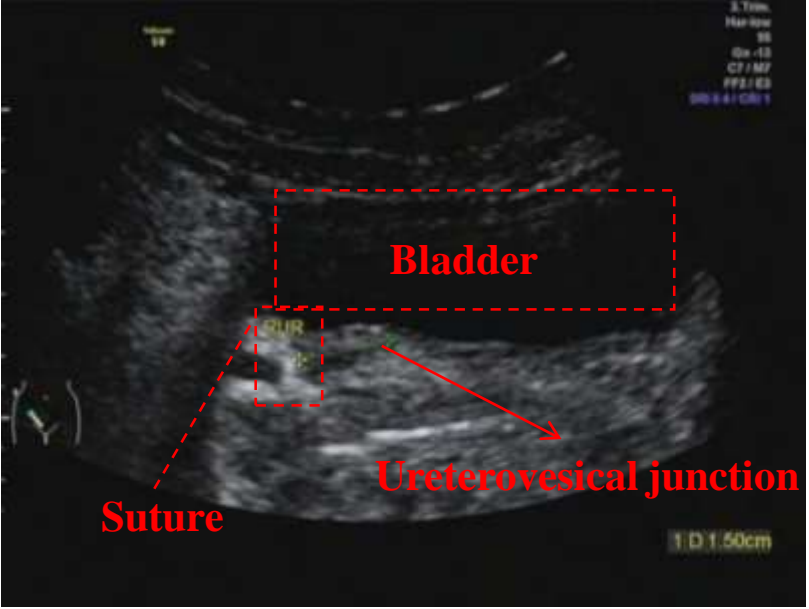

C

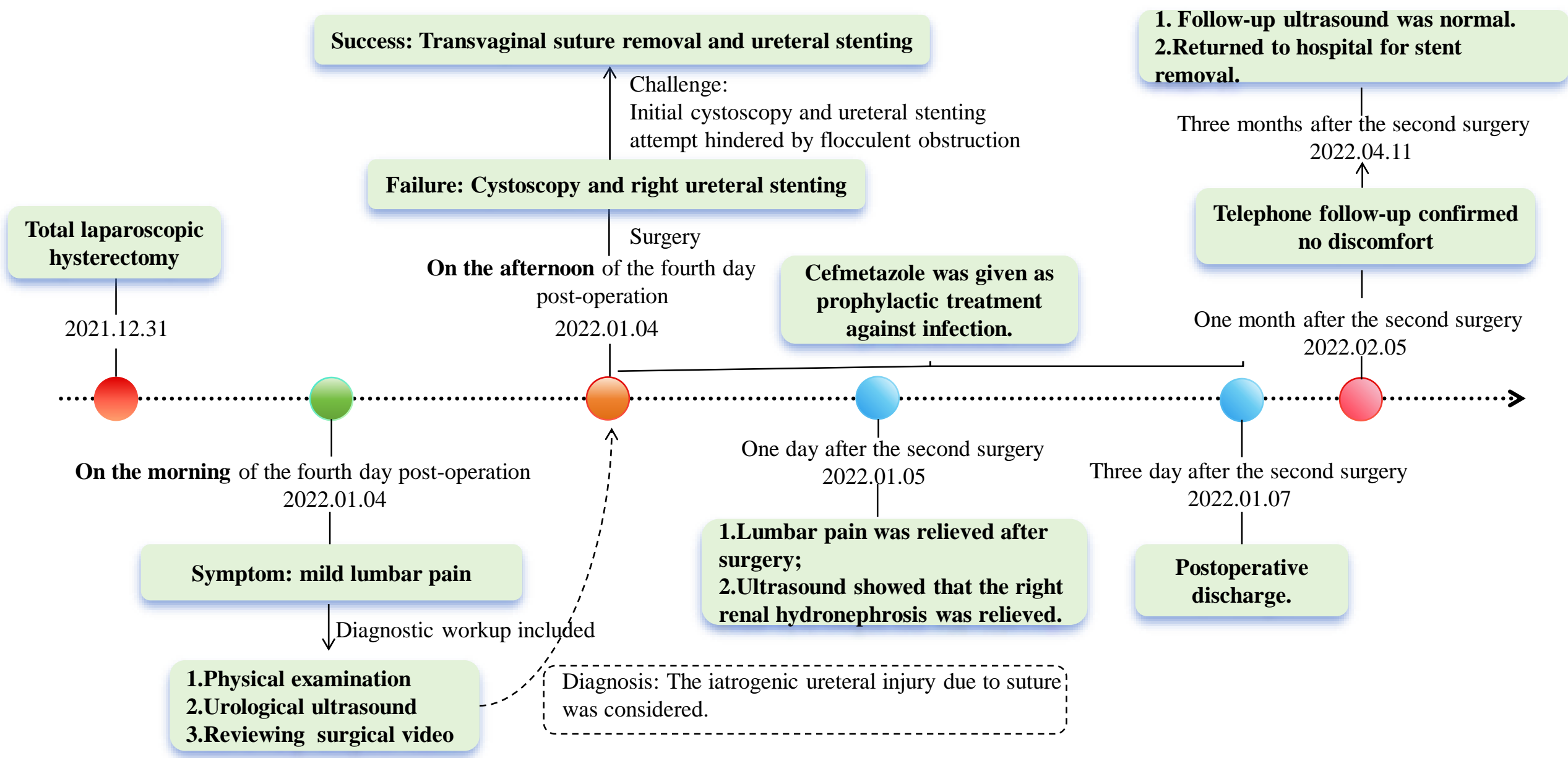

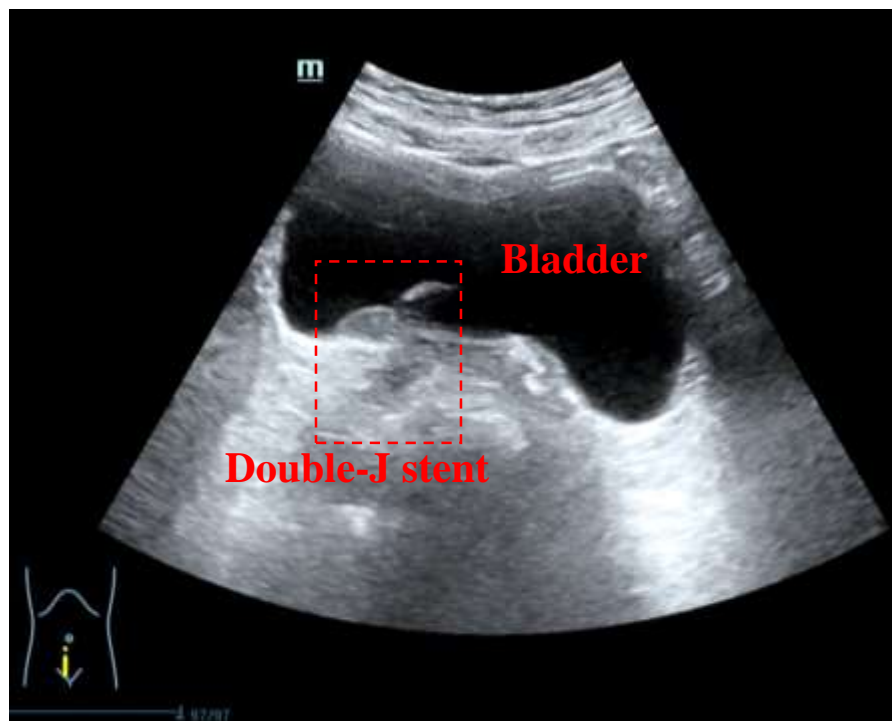

A

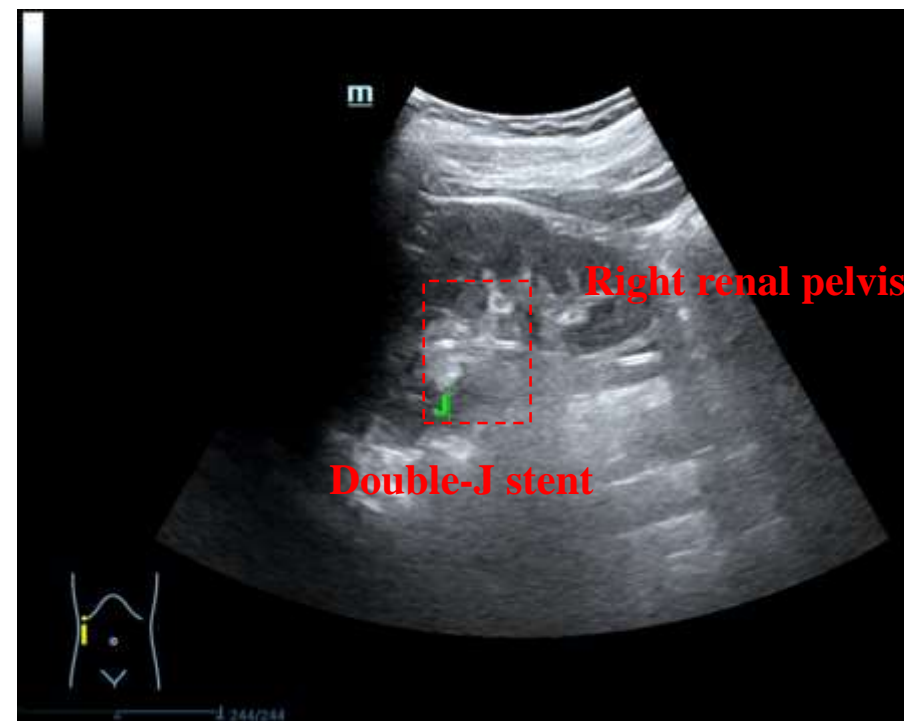

B

Supplement: Supplementary file 1 [file medi-104-e45812-s001.pdf]
